# Specific T-Cell Subsets Can Predict the Efficacy of Anti-TNF Treatment in Inflammatory Bowel Diseases

**DOI:** 10.1007/s00005-020-00575-5

**Published:** 2020-04-04

**Authors:** Sonja Dulic, Gergely Toldi, Florentina Sava, László Kovács, Tamás Molnár, Ágnes Milassin, Klaudia Farkas, Mariann Rutka, Attila Balog

**Affiliations:** 1 Department of Rheumatology and Immunology, Faculty of Medicine, Albert Szent-Györgyi Health Center, University of Szeged, Kálvária sgt. 57, Szeged, 6725 Hungary; 2 First Department of Obstetrics and Gynecology, Semmelweis University, Budapest, Hungary; 3 First Department of Medicine, Faculty of Medicine, Albert Szent-Györgyi Health Center, University of Szeged, Szeged, Hungary

**Keywords:** Th17, Memory T cell, Therapeutic response

## Abstract

The effect of TNF-blockers on T-lymphocyte subsets is largely unknown in inflammatory bowel diseases (IBDs). The aim of the present study was to analyze the prevalence of T-cell subtypes and their correlation to therapeutic response. Sixty-eight patients with Crohn’s disease (CD), 46 with ulcerative colitis (UC) were enrolled. (1) The clinical course was followed after the initiation of TNF-blockers (prospective study). (2) The immunophenotype was also compared between long-term anti-TNF treated-responders and non-responders (cross-sectional study). The results were compared with those of therapy-naïve patients with active disease and those in remission with non-biological immunosuppressive therapy, and with healthy controls. Fourteen subtypes of peripheral blood T cells were measured with flow cytometry. The prevalence of Th2 and Th17 cells, of HLA-DR- and CD69-positive CD4 and CD8 cells, was higher, whereas the percentage of CD45RA-positive CD4 and CD8 cells was lower in both IBDs than in controls. CD8CD69 cell frequency was lower in remission, and decreased during anti-TNF therapy in CD responders. CD8CD45RO memory cells had higher prevalence in UC non-responders than in those starting anti-TNF. CD4CD45RO percentage < 49.05 at the initiation of TNF-blockers was predictive of a subsequent therapeutic response in CD, and Th2 and Th17 prevalence correlated with the duration of remission on TNF-blockers in UC. This study provided a detailed description of the T-cell composition in IBDs. CD8CD69 prevalence may be an activity marker in CD, and CD4CD45RO, Th2 and Th17 levels could be predictive for a therapeutic response to anti-TNF.

## Introduction

Inflammatory bowel diseases (IBDs) are chronic immune-mediated diseases involving the gastrointestinal tract and are frequently associated with extra-intestinal manifestations. IBDs comprise two well-characterized diseases, Crohn’s disease (CD) and ulcerative colitis (UC). The pathogenesis of IBD is still not clearly understood despite intensive research, but an inappropriate mucosal immune response to gut microflora is a well-recognized phenomenon in the pathogenesis (Ogura et al. 2001; Lamas et al. 2016). Both the innate and the adaptive immune system play important roles in the intestinal inflammation. T-lymphocytes comprise a complex collection of highly differentiated T-cell subsets playing key roles in the regulation and the effector phase of the immune response. T-cell phenotype changes have been described in IBD models (Franchi et al. 2017; Zimmermann et al. 2016) and also in human studies (Eastaff-Leung et al. 2010; Mayne and Williams 2013; Yamada et al. 2016; Zhang et al. 2006; Sarra et al. 2010). Decreased numbers of regulatory T cells (Treg) were observed in the circulation and also in the inflamed mucosa (Eastaff-Leung et al. 2010; Mayne and Williams 2013; Yamada et al. 2016), and the importance of Th17 cells is definite as well (Zhang et al. 2006; Sarra et al. 2010). There is an established difference between the two major representative IBD subgroups as CD is associated with Th1 (Fuss et al. 1996), whereas UC is more likely linked with a Th2 predominant profile (Pastorelli et al. 2010; Seidelin et al. 2010, 2015; Tanaka et al. 2010), although this dichotomy is not universal (Wallace et al. 2014).

Biological therapies targeting tumor necrosis factor-α (anti-TNF) are highly effective hallmark therapies in IBD. Despite their widespread use, the impact of TNF-blocking agents on the composition of the adaptive immune system is largely unexplored. In our two previous studies in rheumatoid arthritis (RA) and ankylosing spondylitis (AS), in which anti-TNF therapies are also highly beneficial, we found significant differences in the T-cell subset composition among responder and non-responder patients (Dulic et al. 2017, 2018). Key findings were the normalization of Treg frequencies and the phenotypic pattern of a continuing T-cell activation, as reflected by several activation markers. The prevalence of CD4CD69 cells at the initiation of anti-TNF therapy appeared as a predictive biomarker of a subsequent therapeutic response in RA. Knowledge on such effects in IBDs could clarify the mechanism of action of these therapies in CD and UC, provide information about the status of the adaptive immune system, and could help finding cell-based markers.

## Patients and Methods

To obtain a deeper insight into the changes in the composition of the adaptive immune system during anti-TNF therapy in IBD, and to seek for clinically relevant conclusions, we have separated our study design to two approaches: the first one was a prospective follow-up of CD and UC patients, in whom anti-TNF therapy was initiated. The second one was a cross-sectional comparison of the immune phenotype of IBD patients on established anti-TNF therapy, grouped as anti-TNF-responders and non-responders; these data were compared with results from active, therapy-naïve and inactive (treated) IBD patients. Altogether 114 patients were enrolled. We have measured the prevalence of 14 types of T-cell subsets.

### Patients

CD and UC patients (*n* = 16 for each disease) were enrolled, in whom the disease was active despite therapy with non-biologic immunosuppressive therapies, and in whom anti-TNF therapy was initiated after blood sampling (abbreviated as CDstart or UC start group). All patients had a Crohn’s Disease Activity Index (CDAI) (Best et al. 1976) > 220 (Colombel et al. 2009), or Mayo score > 6 (Schroeder et al. 1987). Patients were classified to disease subsets following the Montreal classification criteria (Satsangi et al. 2006).

During subsequent follow-up, treatment was conducted following the current international guidelines and the best clinical judgement of the treating physician, and data on the subsequent disease activity and therapy were recorded. Short-term (3 months) and long-term (12 months) response to anti-TNF therapy was assessed. Response was defined as a reduction of > 50% in CDAI, or, for UC patients, in the Mayo score, or when endoscopy was not performed at the time of assessment, in the partial Mayo (pMayo) score (Lewis et al. 2008; Sands et al. 2004) without an increase in the anti-TNF dose above standard dose. Non-response (primary or relapse) was established if these activity index-based criteria were not fulfilled, or based on the clinical assessment of the treating physician, corticosteroid had to be introduced, or its dose had to be increased, or anti-TNF dose had to be increased over standard dose, or surgical intervention for active IBD has become necessary, or anti-TNF therapy had to be switched to either another anti-TNF or to a biological of different class. The length of the response period was also recorded. For reimbursement requirements, in some patients at stable remission after 12 months of anti-TNF therapy, the drug was discontinued, and only non-biological maintenance therapy was continued. Disease course after such forced therapy withdrawal was also followed-up. In six patients from each disease group, control blood sampling was performed after at least 3 months of anti-TNF therapy, and the same laboratory examinations were repeated.

As a further assessment, patients on established (> 3 month duration) anti-TNF therapy were also involved. Thirty-one patients with CD and 16 with UC were included, and were distinguished as responders (CDresporUCresp) or non-responders (CDnonrespor UCnonresp) to anti-TNF therapy as defined following the response criteria described in the previous paragraph. Their laboratory results were compared with each other, and with those of the members of CDstart or UCstart groups. The six patients in either the CDstart or UCstart groups, in whom repeated laboratory examinations were performed during anti-TNF treatment, were also involved in the responder or non-responder groups for comparison with the anti-TNF starter groups.

As controls, the following groups were formed and analyzed: (1) patients with newly diagnosed, active, untreated CD or UC: CDnew (*n* = 7), UCnew (*n* = 7); (2) patients with inactive disease controlled with non-biologic immunosuppressive drugs: CDinact (*n* = 14), UCinact (*n* = 7), and (3) age- and sex-matched healthy controls (*n* = 30). All participants gave their informed consent, and the study was conducted in concert with the principles of the Helsinki declaration (ethical approval: ETT-TUKEB 905/PI/09).

### Laboratory Measurements

**Fig. 1:**
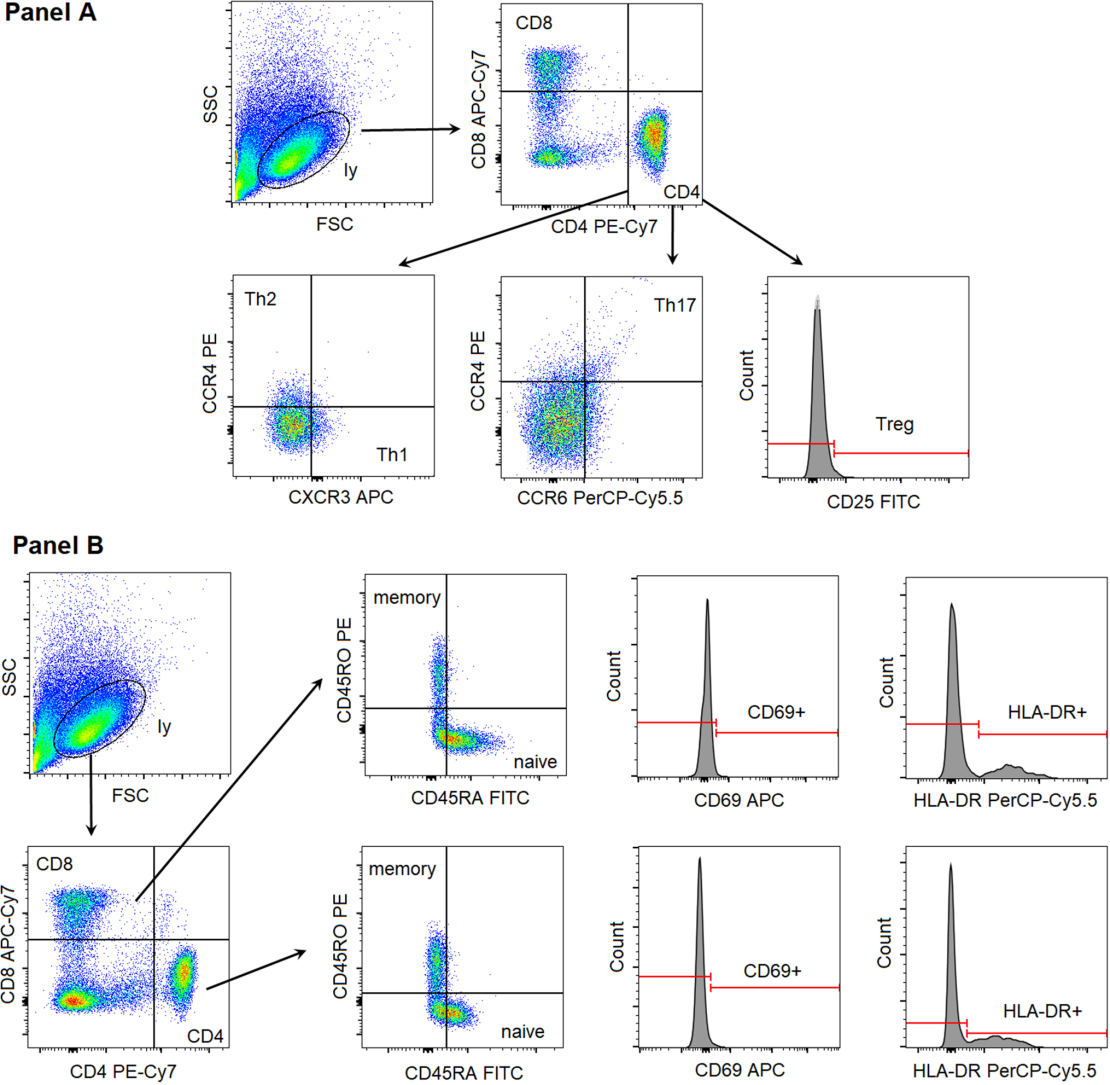
Gating strategy to identify the investigated T-cell subsets. *FSC* forward scatter characteristics, *SSC* side scatter characteristics, *ly* lymphocytes

Fifteen ml of anticoagulated blood samples were taken, and peripheral blood mononuclear cells (PBMCs) were separated by centrifugation with Ficoll-Paque (GE Healthcare Life Sciences, Pittsburgh, PA, USA). Full blood counts revealed that white blood cell and lymphocyte numbers were within normal limits (4–12 G/L and 1–4 G/L, respectively) for all participants. PBMCs were frozen and kept at − 80 °C until analysis. After thawing, samples were washed twice with phosphate-buffered saline solution (pH 7.4). CD3+ T cells were enriched using the EasySep Human T Cell Enrichment Kit (Stemcell Technologies, Cambridge, MA, USA). The following fluorescent antibodies (Becton-Dickinson, San Diego, CA, USA) were applied for cell surface staining: CD4 PE-Cy7 (clone SK3), CD8 APC-Cy7 (clone SK1), CXCR3 APC (clone 1C6/CXCR3), CCR4 PE (clone 1G1), CCR6 PerCP-Cy5.5 (clone 11A9), CD25 FITC (clone M-A251), CD69 APC (clone FN50), HLA-DR PerCP-Cy5.5 (clone G46-6), CD45RA FITC (clone HI100), CD45RO PE (clone UCHL1). T-cell subtypes were differentiated using the following gating strategy (Fig. 1): helper T cells (CD4+CD8−), cytotoxic T cells (CD4−CD8+), Th1 cells (CD4+CXCR3+CCR4−), Th2 cells (CD4+CXCR3−CCR4+), Th17 cells (CD4+CCR4+CCR6+), Tregs (CD4+CD25 high). The proportion of cells expressing the early (CD69), or the late (HLA-DR) activation markers as well as naive T cells (CD45RA+CD45RO−) and memory T cells (CD45RA-CD45RO+) were also determined within both the CD4+ and CD8+ subsets. An average of 200,000 cells was registered for each acquisition. All measurements were performed on a BD FACSAria flow cytometer (BectonDickinson, San Jose, CA, USA). Cell proportion values were determined with conventional gating, through the use of FACSDiva software (BectonDickinson, San Jose, CA, USA).

### Statistical Methods

Clinical data are presented as mean (range), whereas cell subtype prevalence values as mean ± SD or median (25–75 percentile) depending on the distribution of the values. Cell subset percentage values were compared among groups with two-sample *t* test or Mann–Whitney test if two groups were compared, or with analysis of variance or with Kruskal–Wallis test, with Bonferroni’s or Dunn’s tests for multiple comparisons, respectively, if more than two groups were analyzed. We have applied the ANOVA for the comparison of all the ten patient subgroups and healthy controls, and also for the comparison of selected UC or CD patient groups separately, to focus on clinically relevant questions. The patient subgroup analysis according to their therapeutic response or active vs inactive patient subgroups were performed with Student test. Receiver-operated curve (ROC) analyses were performed to seek for values of baseline (i.e., the start of anti-TNF) cell percentages that would discriminate between patients in whom long-term remission was achieved in response to anti-TNF therapy, and Pearson’s correlation analysis was carried out to assess the correlation between cell frequency values at the start of anti-TNF and the length of a subsequent remission. A *p* value < 0.05 was taken statistically significant.

## Results

### Clinical Characteristics and Follow-Up

The demographic and most important clinical data of the patients in all subgroups are summarized in Table 1. Patient subgroups for both diagnoses were similar in terms of age, disease duration, frequency of extraintestinal manifestations, surgical interventions necessitated by IBD, type of anti-TNF, other therapies ever used, or smoking status. Although there were numerical differences among the groups in terms of gender distribution, these were not statistically significant. However, CDinact patients less frequently had perianal manifestations than anti-TNF-treated CD patients (both responders and non-responders), and anti-TNF therapy duration was shorter in UCnonresp patients than in UCresp patients.

**Table 1:** Demographic and clinical data of the different cohorts of IBD patients

	CDstart (*n* = 16)	CDresp (*n* = 23)	CDnonresp (*n* = 8)	CDnew (*n* = 7)	CDinact (*n* = 14)	UCstart (*n* = 16)	UCresp (*n* = 10)	UCnonresp (*n* = 6)	UCnew (*n* = 7)	UCinact (*n* = 7)
Age	30 (18–48)	39 (20–66)	31 (19–48)	31 (25–44)	37 (23–59)	42 (20–65)	40 (19–59)	38 (21–53)	36 (23–48)	43 (26–61)
Female/male	7/9	13/10	3/5	3/4	2/12	7/9	3/7	5/1	3/4	1/6
Disease duration (month)	82 (7–236)	132 (8–410)	121 (17–235)	14 (0–72)	77 (5–204)	105 (6–389)	117 (11–290)	75 (13–180)	3 (1–6)	134 (35–288)
Any extraintestinal manifestation	1	2	0	2	0	2	0	2	1	1
Smoking										
Never	5	9	4	5	9	8	5	3	5	0
Past	1	2	1	0	1	6	2	1	1	3
Present	8	9	3	1	3	1	1	1	1	2
CD Montreal classification										
A1/2/3	2/12/2	3/16/4	1/7/0	0/7/0	3/8/3					
L1/2/3/4	2/2/10/2	2/3/16/2	1/0/7/0	3/2/2/0	2/3/8/1					
B1/2/3	5/6/5	6/9/8	3/2/3	3/2/2	6/6/2					
Perianal Y/N	8/8	16/7	6/2	¾	1/13^**+**^					
UC Montreal classification										
L1/2/3						1/4/11	0/5/5	0/2/4	3/1/3	1/2/4
Current anti-TNF: infliximab/adalimumab		16/7	7/1				8/2	6/0		
Duration of current anti-TNF		18 (4–57	26 (9–62)				11 (4–26)	4 (3–6)*		
Prior anti-TNF	3	9	2			1	5	2		
Corticosteroid (ever)	6	3	3	0	4	10	4	5	0	1
5-ASA/SSZ (ever)	8	4	1	0	3	8	7	1	0	4
AZA (ever)	13	12	6	0	13	12	6	2	0	4
Surgery	7	17	5	0	6	1	1	0	0	0

Numbers indicate mean (range), or number of patients. Extraintestinalmanifestation: musculoskeletal, cutaneous, ocular or hepatobiliary. Prior anti-TNF indicates the number of patients in whom anti-TNF therapy was already applied, but had been stopped at least 12 months before the current therapy was initiated. Smoking habit data were missing in 13 patients

*5-ASA* 5-amino-salycylic acid, *SSZ* sulfasalazine, *AZA* azathioprine. For the explanation of the abbreviations of patient subgroups, please see the “Patients” section

^**+**^*p* < 0.05 for comparison of CDinact with CDresp and CDnonresp;*p < 0.05 for comparison of UCresp vs UCnonresp

The mean follow-up time of the patients after the blood sampling and the initiation of anti-TNF therapy was 35 months for CD, and 24 months for UC patients. Follow-up data are summarized in Table 2. Long-term therapeutic response to anti-TNF was observed in 14 of 16 CD patients and 9 of 16 UC patients. Primary non-response occurred slightly more often than loss of effect—secondary inefficacy—in anti-TNF-refractory UC patients. The disease relapsed within 12 months in 9 of the 11 patients in whom anti-TNF was stopped after 1 year of successful therapy, and in only 2 of them was a biologic-free remission observed, during a follow-up period of 12 and 36 months, respectively.

**Table 2:** Disease outcome after the initiation of anti-TNF therapy in IBD patients

	CD (*n* = 16)	UC (*n* = 16)
Duration of follow-up (months)	35 (16–47)	24 (2–46)
Short-term response achieved	15	12
Long-term response achieved	14	9
Duration of response	27 (0–47)	19 (0–46)

For 4 UC patients, follow-up was shorter than 12 months, long-term response was, therefore, evaluable in 20 patients. For the definition of short-term and long-term response, see Patients section

### Lymphocyte Subset Distribution

**Fig. 2:**
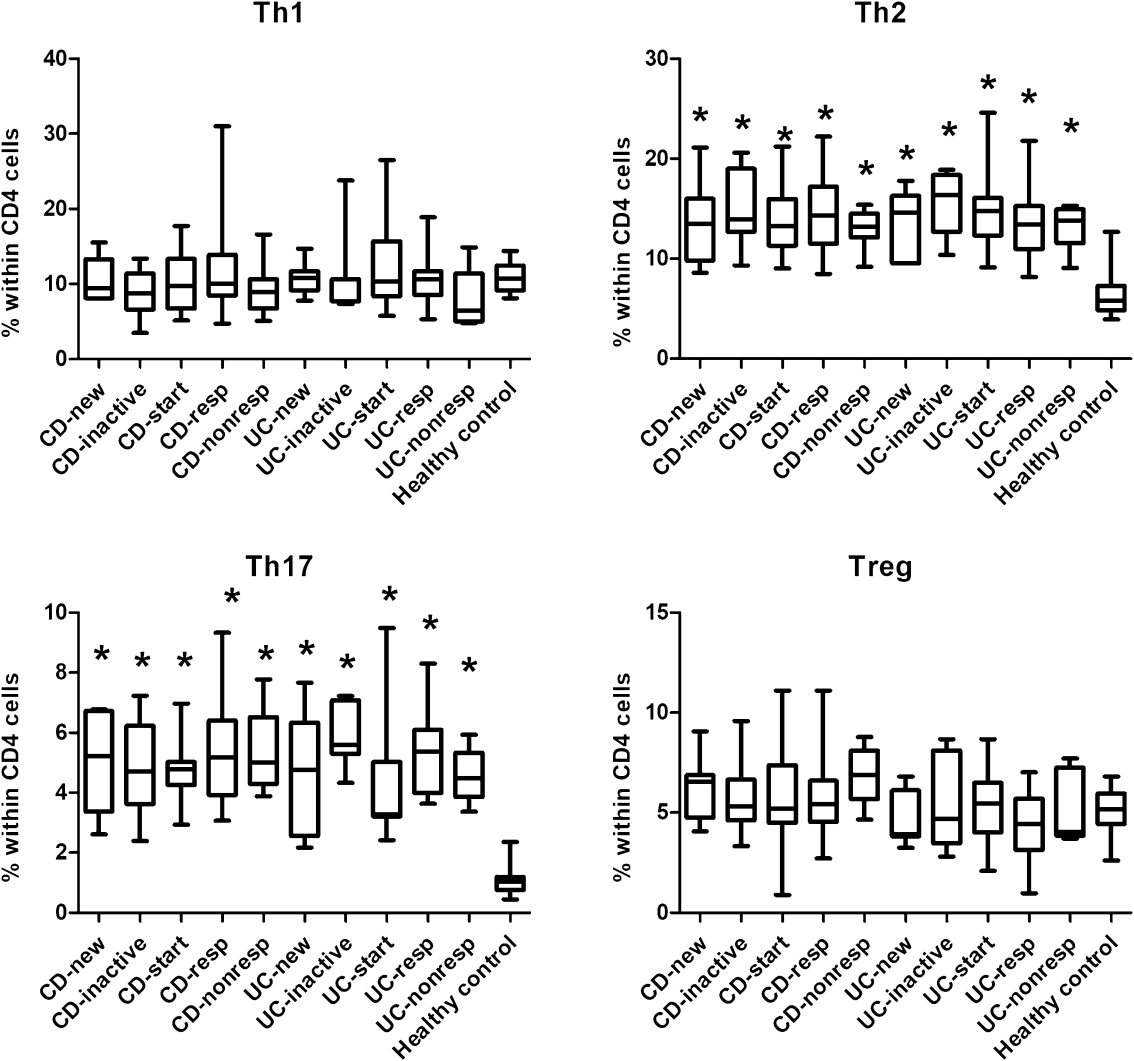
Comparison of the frequencies of the major T helper cell subsets among the various patient groups and controls. *CDnew* new-onset Crohn’s disease (CD) patients, *CDinactive* CD patients with inactive disease on immunosuppressive therapy, *CDstart* patients with active CD in whom anti-TNF therapy is initiated, *CDresp* anti-TNF-responder CD patients, *CDnonresp* anti-TNF-nonresponder CD patients. Abbreviations are analogous for ulcerative colitis (UC) patients. Data are presented as median (horizontal line within boxes), 25 and 75 percentile (horizontal borders of the boxes), and minimum and maximum (whiskers). Numbers on the vertical axis indicate % within CD4 cells. *p < 0.05 vs healthy controls with ANOVA and Bonferroni’s correction

**Fig. 3:**
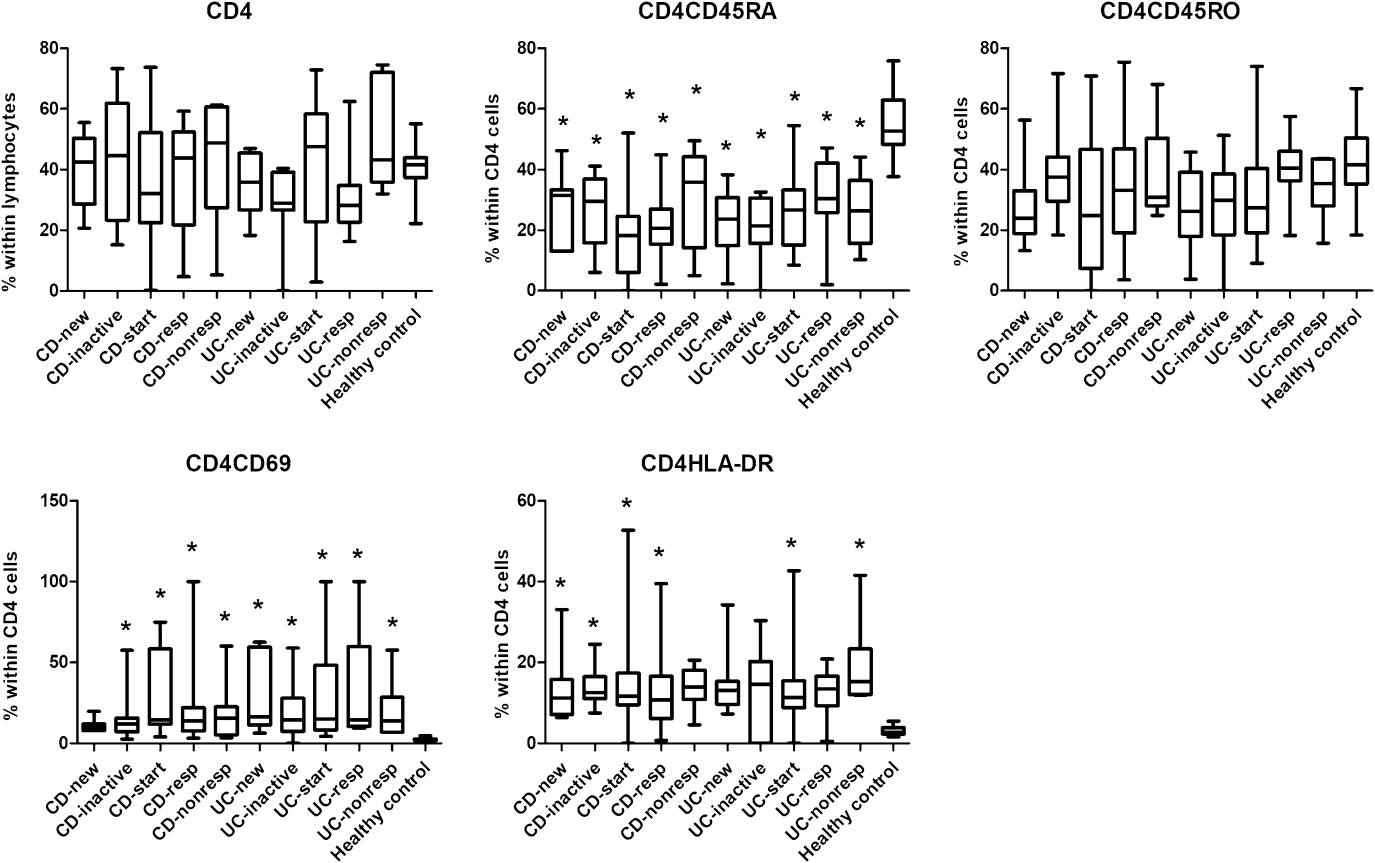
Comparison of the frequencies of CD4 cell subsets among the various patient groups and controls. *CDnew* new-onset Crohn’s disease (CD) patients, *CDinactive* CD patients with inactive disease on immunosuppressive therapy, *CDstart* patients with active CD in whom anti-TNF therapy is initiated, *CDresp* anti-TNF-responder CD patients, *CDnonresp* anti-TNF-nonresponder CD patients. Abbreviations are analogous for ulcerative colitis (UC) patients. Data are presented as median (horizontal line within boxes), 25 and 75 percentile (horizontal borders of the boxes), and minimum and maximum (whiskers). Numbers on the vertical axis indicate % within CD4 cells. *p < 0.05 vs healthy controls with ANOVA and Bonferroni’s correction

**Fig. 4:**
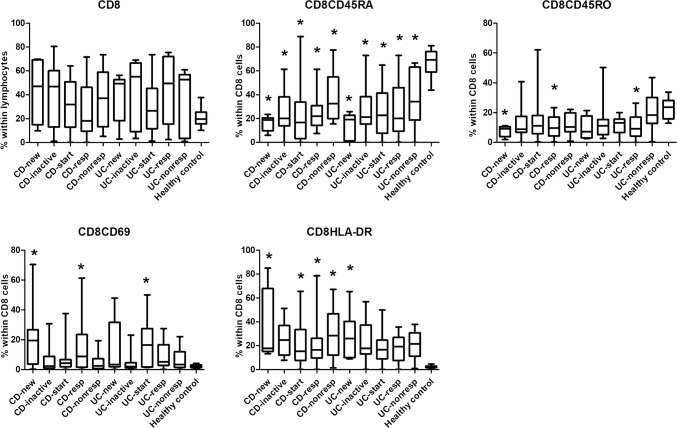
Comparison of the frequencies of CD8 cell subsets among the various patient groups and controls. *CDnew* new-onset Crohn’s disease (CD) patients, *CDinactive* CD patients with inactive disease on immunosuppressive therapy, *CDstart* patients with active CD in whom anti-TNF therapy is initiated, *CDresp* anti-TNF-responder CD patients, *CDnonresp* anti-TNF-nonresponder CD patients. Abbreviations are analogous for ulcerative colitis (UC) patients. Data are presented as median (horizontal line within boxes), 25 and 75 percentile (horizontal borders of the boxes), and minimum and maximum (whiskers). Numbers on the vertical axis indicate % within CD4 cells. *p < 0.05 vs healthy controls with ANOVA and Bonferroni’s correction

We have revealed a number of significant differences between healthy controls and IBD patients with respect of the prevalences of several T-cell subsets. Every CD or UC subgroup had higher frequencies of Th2 and Th17 cells (Fig. 2) than healthy controls. Although the proportion of CD4 cells was comparable across all groups, patients in all IBD subgroups had less naive (CD45RA+) T cells than healthy controls, whereas the proportion of activated (CD69+ and HLA-DR+) T cells was higher in most IBD subgroups, especially in CD patients, than in healthy controls (Fig. 3). In the CD8 compartment, similar conclusions can be drawn, with the addition that the prevalence of memory T cells (CD45RO+) proved to be statistically significantly higher in 2 CD and 1 UC subgroup too (Fig. 4).

**Fig. 5:**
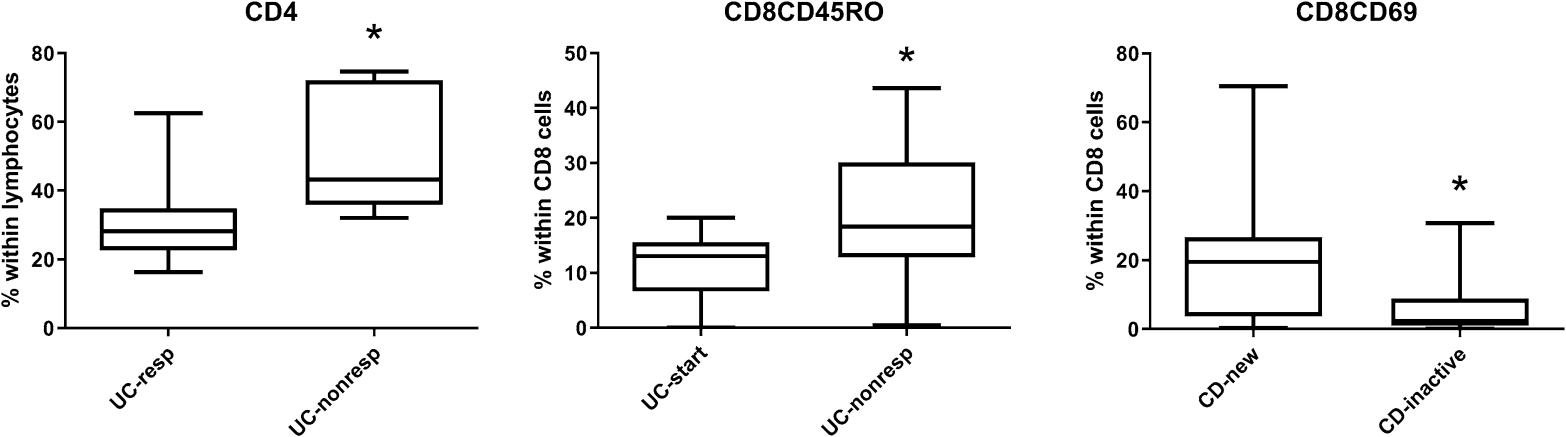
Statistically significant differences between particular patient subgroups. *CDnew* new-onset Crohn’s disease (CD) patients, *CDinactive* CD patients with inactive disease on immunosuppressive therapy, *CDstart* patients with active CD in whom anti-TNF therapy is initiated, *CDresp* anti-TNF-responder CD patients, *CDnonresp* anti-TNF-nonresponder CD patients. Abbreviations are analogous for ulcerative colitis (UC) patients. Data are presented as median (horizontal line within boxes), 25 and 75 percentile (horizontal borders of the boxes), and minimum and maximum (whiskers). Numbers on the vertical axis indicate % within CD4 cells. *p < 0.05 with Student *t* test

However, in contrast with our previous studies on RA and AS patients, relatively few differences could be demonstrated between newly diagnosed cases and patients in remission, and among patients before and after the initiation of anti-TNF therapy. Significant differences among the various patient subgroups based on their therapeutic response in the cross-sectional part are summarized in Fig. 5, and are as follows: CD4 proportion was lower in anti-TNF responder UC patients than in non-responders (31 ± 4.3 vs 50 ± 7.4, *p* < 0.05); however, there was no significant difference between CDresp and CDnonresp groups. CD8CD69 activated T cells were observed more frequently in newly diagnosed CD patients than in those whose disease has become inactive with conventional immunosuppressive therapy (21.15 ± 9.13 vs 6.39 ± 2.23, *p* < 0.05); and CD8 memory cell prevalence was higher in anti-TNF non-responder UC patients than in UC patients at the time of anti-TNF-initiation (20.57 ± 5.73 vs 11.40 ± 1.49, *p* < 0.05), whereas this parameter did not change significantly in anti-TNF responder UC patients. In contrast with our previous findings in RA and AS, Treg prevalence was not affected by anti-TNF therapy in either IBD.

Finally, we analyzed if any of the cell prevalence values had a predictive value for the efficacy of anti-TNF therapy. We performed ROC analyses in the CDstart and UCstart groups to search for frequency values that could discriminate between patients with and without long-term remission, and we carried out correlation analysis between the cell percentages and the duration of remission. In CD patients, CD4CD45RO cell prevalence of < 49.05% has predicted the long-term remission with a sensitivity of 100%, and a specificity of 92.3%, area under the curve (AUC): 1.0 (*p* = 0.03). CD4HLADR cell frequencies showed a near-significant (*p* = 0.058) correlation with the duration of remission (Pearson’s *r* = 0.50). In UC patients, there was a trend to significance (*p* = 0.08) for a CD4 prevalence value of < 29.95 (sensitivity 66.67%, specificity 87.50%, AUC 0.85), and Th2 and Th17 cell prevalence values showed a significant positive correlation with the duration of response (*p* = 0.03, Pearson’s *r* 0.59, and *p* = 0.03, Pearson’s *r* 0.61, respectively), and a tendency with regard to a positive correlation with CD8HLADR (*p* = 0.06, Pearson’s *r* 0.59).

## Discussion

The present study has provided a detailed characterization of the adaptive immune system in CD and UC, in particular, the prevalence of several T-cell subtypes that have not been studied before. Elevated frequencies of memory and activated T cells and decreased proportion of naïve T cells have been revealed in both diseases. Th17 cells were found to be expanded as compared with controls in both IBDs as reported before (Zhang et al. 2006; Sarra et al. 2010). The finding of elevated Th2 cell percentages in UC is also in line with the majority of published results (Pastorelli et al. 2010; Seidelin et al. 2010, 2015; Tanaka et al. 2010), but in contrast with the previously presumed predominance of Th1 over Th2 in CD, according to our findings, Th1 cells have the same ratios in both CD and UC as those in healthy controls. The universal role of the Th1 skewing of T-cell differentiation has been questioned by some investigators (Wallace et al. 2014; Niessner and Volk 1995; Rovedatti et al. 2009), and the lack of a convincing benefit of monoclonal antibody treatment targeting interferon-γ in CD (Cui et al. 2013) can also argue against the importance of this pathway. Furthermore, differences in disease activity, disease stage and patient population from the published examinations may also explain these discrepancies.

The most remarkable finding is that CD8CD69-positive T-cell prevalence is an activity biomarker, because it is more abundant in active CD patients than in those in remission. Therefore, low CD8CD69 prevalence may confirm that the disease is in remission. This is in line with earlier literature highlighting the importance of CD69 in inflammatory bowel disease (Radulovic and Niess 2015). Of note, in some of our patients, the prevalence of circulating CD69+ cells was higher than expected, as these cells normally reside in the mucosa, interacting with the microbiome. Their egression into the periphery may indicate the importance of other circulating antigens in the pathogenesis of IBD. Furthermore, spondylarthropathic disorders, including psoriatic arthritis have also been recently characterized by an increased frequency of circulating CD8+ CD69+ T cells compared to psoriasis and healthy controls (Diani et al. 2019).

Lower than 49.05% prevalence of CD4CD45RO is a further putative biomarker, as it is predictive for long-term treatment response, but only in CD. A higher percentage of CD4HLADR in CD, and a higher frequency of Th2 and Th17 cells in UC can also predict a longer duration of remission. The importance of Th17 prevalence is also confirmed by the studies of Hull et al., who found that Th17 cell frequency increases during anti-TNF therapy in RA, AS and psoriatic arthritis, and the increase is associated with the improvement of synovitis in RA (Hull et al. 2015, 2016).

Another goal of this examination was the analysis of changes in the circulating T-cell composition during anti-TNF therapy. In contrast with our similar studies on RA and AS patients, we have found very little changes in the T-cell subset distribution when anti-TNF-treated and treatment-naïve patients were compared, and the T-cell profile did not differ markedly between anti-TNF-responders and non-responders. Our primary hypothesis for this finding is that in IBDs the most relevant immunological events happen in the gut mucosa-associated immune system, and are reflected less in the circulating lymphocyte pool than in RA and AS. We propose, therefore, that studies on the pharmacodynamic immunological effects of anti-TNF, and possibly other novel targeted therapies are more appropriate from mucosal biopsies than from peripheral blood, or parallel samplings from endoscopic biopsies and peripheral blood should be performed. We note that peripheral blood T-cell composition did prove to be useful for the description of the adaptive immune system when CD and UC patients were compared with healthy individuals, and it may be possible that an even longer follow-up would be needed until the putative mucosal immune alterations would proceed to the systemic immune system.

In summary, several candidate cell-based biomarkers have emerged: CD8CD69 in both IBDs, CD4CD45RO and CD4HLADR in CD, and CD4, Th2, Th17 and CD8HLADR in UC all seem to be promising markers of remission or of the prediction of a therapeutic response to anti-TNF. Since only 40–50% of patients achieve a good and sustained reaction to anti-TNF treatment (Bálint et al. 2016), and the cause of the failure was primary non-response in most of our cases, validation of these finding would provide us with tools for a more personalized choice from the growing number of classes of targeted therapies in IBD.
